# Ultrasound-Based Staging and Its Impact on Clinical Management of Hepatic Hydatid Cysts in an Endemic Setting: A Cross-Sectional Study in Eastern Afghanistan

**DOI:** 10.3390/tropicalmed11070172

**Published:** 2026-06-24

**Authors:** Samiullah Sajjad, Parnpen Viriyavejakul, Dorn Watthanakulpanich, Sant Muangnoicharoen, Paron Dekumyoy, Wirongrong Chierakul, Chayasin Mansaguan, Prakaykaew Charunwatthana

**Affiliations:** 1 Department of Clinical Tropical Medicine, Faculty of Tropical Medicine, Mahidol University, Bangkok 10400, Thailand; dsajjad411@gmail.com (S.S.); sant.mua@mahidol.edu (S.M.); wirongrong.chi@mahidol.ac.th (W.C.); chayasin.man@mahidol.ac.th (C.M.); 2 Department of Medicine, Nangarhar Medical Faculty, Nangarhar University, Jalalabad 2601, Afghanistan; 3 Department of Tropical Pathology, Faculty of Tropical Medicine, Mahidol University, Bangkok 10400, Thailand; parnpen.vir@mahidol.ac.th; 4 Department of Helminthology, Faculty of Tropical Medicine, Mahidol University, Bangkok 10400, Thailand; dorn.wat@mahidol.ac.th (D.W.); paron.dek@mahidol.ac.th (P.D.); 5 Mahidol-Oxford Research Unit, Faculty of Tropical Medicine, Mahidol University, Bangkok 10400, Thailand

**Keywords:** *Echinococcus granulosus*, hydatid cyst, imaging modalities, ultrasound, computed tomography, case management

## Abstract

**Background:** Hydatid disease, caused by *Echinococcus granulosus*, remains a significant public health concern in endemic regions. This study aimed to evaluate the role of ultrasound in the diagnosis, staging, and clinical management of liver hydatid cysts in the eastern city of Jalalabad, Afghanistan. **Method:** A cross-sectional study was conducted between February and November 2024 among 159 patients diagnosed with liver hydatid cysts. Demographic, clinical, laboratory, and imaging data were collected. Cysts were classified according to the WHO Informal Working Group on Echinococcosis (WHO-IWGE) and Gharbi systems. Ultrasound findings were compared with computed tomography (CT), and their association with treatment decisions was assessed. **Result:** A total of 159 patients with liver hydatid cysts were included in the study. Among them, 91 (57.2%) were female, 80 (50.3%) were aged 20–39 years, and 128 (80.5%) resided in rural areas. Most patients presented with a single cyst (144/159, 90.6%), while multiple cysts were observed in 15 (9.4%). The majority of cysts measured 5–9.9 cm in diameter (43.4%), followed by 1–4.9 cm (42.1%) and ≥10 cm (14.5%). According to the WHO-IWGE classification, CE1 (25.8%) and CE4 (24.5%) were the most common stages, followed by CE2 (17.6%), CE3a (13.8%), CE3b (11.3%), and CE5 (7.0%). Common exposure-related factors included dog ownership, poor hygiene practices, and consumption of raw vegetables. Ultrasound accurately identified cyst stages and demonstrated a significant association between WHO-IWGE staging and treatment modality (χ^2^ = 63.56, *p* < 0.001). Almost perfect agreement was observed between ultrasound and CT for cyst classification (Cohen’s κ > 0.90), although CT provided additional anatomical information in selected complex cases. **Conclusions:** Ultrasound is an accessible, accurate, and reliable imaging modality for the diagnosis, staging, and management of liver hydatid cysts. In resource-limited settings, it serves as the primary imaging modality for guiding clinical decision-making, with CT reserved for complex or uncertain cases.

## 1. Introduction

Cystic echinococcosis (CE), or hydatid disease, is a parasitic zoonosis caused by the larval stage of *Echinococcus granulosus*. It poses serious health risks due to complications such as cyst rupture, secondary infection, or pressure effects on adjacent organs, although up to 60% of patients may remain asymptomatic for extended periods [1]. Two main forms are recognized: cystic echinococcosis, caused by *E. granulosus*, and alveolar echinococcosis (AE), caused by *E. multilocularis* [2].

Globally, CE remains highly endemic in several regions, particularly in rural and livestock-dependent areas. Prevalence rates of 5–10% have been reported in parts of Argentina, Peru, East Africa, Central Asia, and China, with incidence levels exceeding 50 cases per 100,000 person-years in some settings [3]. In South America, animal infection rates in slaughterhouses have ranged between 20% and 95%, highlighting the persistence of the zoonotic transmission cycle in these regions [3]. The World Health Organization (WHO) estimates that echinococcosis affects over one million individuals annually, causing approximately 19,300 deaths in 2015 and generating an economic burden of nearly USD 3 billion each year due to healthcare costs and livestock losses [4].

The diagnosis of hepatic CE primarily relies on imaging modalities. Ultrasound (US) is widely considered the gold-standard tool, providing reliable visualization of cyst morphology and activity. The WHO Informal Working Group on Echinococcosis (WHO-IWGE) and Gharbi classifications standardize ultrasound findings to guide management decisions [5,6]. Other imaging techniques, such as computed tomography (CT) and magnetic resonance imaging (MRI), serve as complementary methods, particularly in complex or atypical cases. Emerging modalities, including contrast-enhanced ultrasound (CEUS), shear wave elastography (SWE), and positron emission tomography (PET), offer additional diagnostic potential, although they remain less accessible in endemic, resource-limited settings [7].

Several imaging-based classification systems have been developed to standardize the evaluation of hydatid cysts. The Gharbi classification categorizes cysts into five sonographic types according to their morphological appearance, whereas the WHO Informal Working Group on Echinococcosis (WHO-IWGE) classification categorizes cysts into active (CE1–CE2), transitional (CE3), and inactive (CE4–CE5) stages based on biological activity and viability. The WHO-IWGE system is currently recommended for guiding treatment selection and follow-up of cystic echinococcosis [8,9,10].

Management of CE depends on cyst stage and includes medical therapy (e.g., albendazole), percutaneous interventions, surgery, or conservative “watch-and-wait” approaches. Conventional surgical treatment carries a mortality rate of 0.9–3.6% and a recurrence risk of approximately 11.3% within the first five years [11].

Recent evidence from neighboring endemic regions, including Khyber Pakhtunkhwa, Pakistan, continues to demonstrate the persistence of cystic echinococcosis in livestock populations, highlighting the ongoing public health and economic importance of the disease in South and Central Asia [12].

Although several studies have evaluated the role of ultrasonography and computed tomography in the diagnosis of cystic echinococcosis, evidence from Afghanistan remains extremely limited. Most published studies originate from other endemic regions and may not fully reflect the epidemiological, clinical, and healthcare characteristics of eastern Afghanistan. In addition, few studies have simultaneously assessed imaging findings using both the WHO-IWGE and Gharbi classification systems within a routine clinical setting. The present study provides locally generated evidence from an endemic area, evaluates the practical diagnostic performance of ultrasonography and CT in real-world conditions, and describes the distribution of cyst stages among affected patients. These findings may contribute to improved diagnosis, staging, clinical management, and public health planning for hydatid disease in resource-limited endemic settings.

## 2. Methods

### 2.1. Study Design, Study Site and Period

This cross-sectional study was conducted in Jalalabad City, Afghanistan, over a nine-month period from 13 February to 16 November 2024, following ethical approval.

### 2.2. Participants and Eligibility

The study population comprised patients with suspected liver hydatid cysts. Individuals of all ages and both sexes were eligible for inclusion if they were diagnosed with liver hydatid cysts by qualified sonologists and provided written informed consent to participate. Patients were excluded if they had a prior history of liver hydatid cyst surgery, were receiving ongoing medical treatment for the disease, were diagnosed with hepatic alveolar echinococcosis, or were pregnant at the time of enrolment.

### 2.3. Sample Size

A total of 159 patients with liver hydatid cysts were enrolled in the study. Participants were recruited consecutively during the study period based on case availability in the study setting. Although an initial sample size estimation was performed using Epi Info statistical software, assuming a 95% confidence level, 80% power, 5% margin of error, and an expected prevalence (P) of 10%, yielding a minimum required sample size of 139, the final sample size was determined pragmatically by the total number of eligible patients identified during the study period.

### 2.4. Data Collection and Study Procedure

Patients presenting with abdominal or pelvic symptoms to public or private ultrasound centers were initially evaluated by local sonologists trained in cystic echinococcosis (CE) staging. Suspected cases of liver hydatid cysts were referred to Daudzai Medical Complex, a private diagnostic and referral facility.

After providing detailed information about the study, written informed consent was obtained from each participant by the principal investigator and trained study personnel. Following consent, demographic, socioeconomic, exposure-related, and clinical data were collected using a structured case record form. Each enrolled patient underwent repeat ultrasonography performed by an experienced radiologist using standardized liver imaging protocols. In addition, abdominal computed tomography (CT) was conducted to further characterize cyst features, and serological testing (ELISA) for cystic echinococcosis was performed. Participant recruitment continued until the target sample size was reached.

### 2.5. Laboratory Procedures

Venous blood (5 mL) was collected from each participant during the initial clinical evaluation prior to treatment initiation. Blood samples were obtained by trained laboratory personnel using a sterile disposable 5 mL syringe fitted with a 21-gauge needle under aseptic conditions. The venipuncture site was disinfected with 70% isopropyl alcohol and allowed to air dry before needle insertion. Blood was collected from a peripheral vein, and specimens were immediately transferred into plain tubes for serological testing and EDTA tubes for hematological investigations. Serum was separated by centrifugation at 3000 rpm for 10 min and stored at 2–8 °C until serological analysis. All blood collection and laboratory procedures were performed according to standard operating protocols at Daudzai Medical Complex laboratory.

*Echinococcus granulosus*-specific IgG antibodies were detected using the DRG Echinococcus IgG ELISA kit (DRG Instruments GmbH, Marburg, Germany), following the manufacturer’s instructions. The assay has a reported sensitivity of 90–95% and specificity of 95–98% for hepatic hydatid disease.

Complete blood count and liver function tests were performed for all patients to assess eosinophilia and hepatic involvement. Although ELISA provides high diagnostic accuracy, false-negative results may occur in inactive cysts, and false-positive results may arise due to cross-reactivity with other parasitic infections. Therefore, serological findings were interpreted in conjunction with imaging results.

### 2.6. Abdominal Imaging and Treatment Approach

Abdominal ultrasonography was performed using a Nemio MX ultrasound system (Toshiba Medical Systems Corporation, Otawara, Tochigi, Japan) equipped with a convex abdominal transducer (3.5–5.0 MHz). All examinations were performed by experienced radiologists according to standard imaging protocols. Cysts were classified according to both the Gharbi (Types I–V) and WHO Informal Working Group on Echinococcosis (WHO-IWGE; CE1–CE5) classification systems. Computed tomography (CT) scans were performed to confirm ultrasound findings and to further assess cyst calcification, septation, and involvement of adjacent organs. Imaging findings were independently reviewed by radiologists who were blinded to the patients’ clinical data.

Treatment decisions were individualized according to cyst stage, clinical presentation, and available healthcare resources. Inactive cysts (CE4–CE5) were managed conservatively using a “watch-and-wait” approach, while albendazole (400 mg twice daily in 28-day cycles) was prescribed for active cysts (CE1–CE3a) or as peri-interventional therapy. Selected CE1 and CE3a cysts larger than 5 cm underwent PAIR (puncture, aspiration, injection, and re-aspiration) under ultrasound guidance, whereas surgical intervention was reserved for complicated, large, or refractory cysts. All patients underwent follow-up assessment through repeat ultrasonography and clinical evaluation to monitor treatment response and detect possible recurrence.

### 2.7. Outcomes and Definitions

The primary outcome of this study was to evaluate the clinical utility of ultrasound-based staging, according to the WHO Informal Working Group on Echinococcosis (WHO-IWGE) classification, in guiding the management of liver hydatid cysts. This included assessment of the relationship between cyst stage, clinical and laboratory characteristics, and treatment approaches (medical therapy, PAIR, surgery, or conservative management).

Secondary outcomes included comparison of cyst staging between ultrasound and computed tomography (CT), as well as documentation of post-treatment complications and factors associated with treatment outcomes.

For the purposes of this study, treatment outcome was defined as the clinical and imaging response following management and was categorized as improved (resolution or regression of cysts without complications) or not improved (persistent cyst activity, recurrence, or complications). Complications were defined as adverse events such as cyst rupture, infection, biliary communication, or recurrence identified during follow-up evaluation.

Ultrasound-based staging was performed according to the WHO-IWGE classification system (CE1–CE5), in which CE1–CE3a cysts were classified as active, CE3b cysts as transitional, and CE4–CE5 cysts as inactive.

### 2.8. Statistical Analysis

Data were analyzed using IBM SPSS Statistics version 26.0 (IBM Corp., Armonk, NY, USA). Descriptive statistics were used to summarize socio-demographic characteristics, clinical features, laboratory findings, imaging results, and treatment modalities. Categorical variables were presented as frequencies and percentages.

The Chi-square test was used to assess associations between cyst stage based on the WHO ultrasound classification, treatment modality, and clinical outcomes. A *p*-value < 0.05 was considered statistically significant. Due to the small sample size in certain treatment categories, the results of subgroup analyses were interpreted with caution. When the expected frequency in contingency table cells was less than five, Fisher’s exact test was used instead of the Chi-square test.

Agreement between ultrasound and computed tomography (CT) findings according to the Gharbi and WHO classifications was evaluated using Cohen’s Kappa coefficient and interpreted according to standard criteria as follows: slight (0.01–0.20), fair (0.21–0.40), moderate (0.41–0.60), substantial (0.61–0.80), and almost perfect agreement (>0.80).

These analyses were performed to evaluate the role of ultrasound in the staging and management of liver hydatid cysts.

### 2.9. Ethical Considerations and Consent to Participate

The study received ethical approval from the Institutional Review Board of Nangarhar Medical Faculty, Afghanistan (NMF REC 013-2023), and the Ethics Committee of the Faculty of Tropical Medicine, Mahidol University (MUTM 2024-002-01). Before enrolment, trained interviewers explained the study objectives, procedures, and potential risks and benefits to each participant. Written informed consent was obtained from all participants prior to data collection and diagnostic evaluation. All study procedures were conducted in accordance with the principles of the Declaration of Helsinki.

## 3. Result

### 3.1. Study Profile and Baseline Characteristics

A total of 159 patients with liver hydatid cysts were included in the study. Among them, 91 (57.2%) were female, 80 (50.3%) were aged 20–39 years, and 128 (80.5%) resided in rural areas. Most patients presented with a single cyst (144/159, 90.6%), while multiple cysts were observed in 15 (9.4%). The majority of cysts measured 5–9.9 cm in diameter (43.4%), followed by 1–4.9 cm (42.1%) and ≥10 cm (14.5%). Detailed demographic and socioeconomic characteristics are presented in Table 1.

### 3.2. Clinical and Laboratory Findings

Overall, 43.4% of patients presented with clinical symptoms. The most frequently reported symptoms were abdominal pain (42.8%), fever (29.0%), nausea/vomiting (23.3%), and abdominal distension (15.1%), whereas jaundice (3.1%) and weight loss (9.4%) were less common. Physical examination findings included abdominal tenderness (42.1%), hepatomegaly (12.6%), and ascites (0.6%). The single case of ascites was associated with advanced hepatic involvement and was considered clinically related to complicated liver hydatid disease.

Laboratory findings demonstrated anemia in 28.9% of patients, leukocytosis in 14.5%, eosinophilia in 22.6%, elevated bilirubin levels in 6.3%, and increased alkaline phosphatase levels in 22.6%, suggesting parasitic infection and hepatic involvement. Stool examinations detected intestinal parasites in 30.8% of participants, while ELISA serology was positive in 66.0% of cases (Table 2).

### 3.3. Symptom Duration According to WHO Cyst Stage

Analysis of symptom duration according to WHO cyst stage demonstrated a relationship between disease stage and chronicity. Patients with early-stage cysts (CE1 and CE2) most commonly presented within 8–30 days of symptom onset (69.6% and 70.6%, respectively). In contrast, transitional and advanced stages were associated with longer symptom duration. Most CE3a cases were observed within 31–90 days (61.5%), whereas CE3b cases were predominantly associated with symptoms lasting longer than 90 days (80.0%). All CE4 cases were associated with symptom duration exceeding 90 days. No symptomatic CE5 patients were identified during the study period (Table 3).

Overall, 44.9% of patients presented within 8–30 days of symptom onset, 24.6% within 31–90 days, 7.2% within 0–7 days, and 23.2% after more than 90 days. These findings suggest progressive chronicity with advancing cyst stage.

### 3.4. Association Between ELISA Serology and WHO Cyst Stage

The distribution of ELISA serology results across WHO cyst stages demonstrated a significant association between cyst activity and antibody detection. Seropositivity was highest in active cyst stages, including CE1 (97.6%) and CE2 (89.3%), while transitional stages showed intermediate positivity rates, including CE3a (72.7%) and CE3b (72.2%). Lower seropositivity was observed in inactive stages, including CE4 (18.0%) and CE5 (36.4%) (Table 4).

Overall, 66.0% of patients were ELISA positive, whereas 34.0% were negative. The association between WHO cyst stage and ELISA results was statistically significant (*p* < 0.001). The relatively higher seropositivity observed in CE5 compared with CE4 may reflect persistent antibody responses despite cyst calcification.

### 3.5. Treatment Modalities and Clinical Characteristics

The most common treatment approaches were “watch-and-wait” management (30.8%), surgery combined with medication (29.6%), and PAIR combined with medication (25.2%). Medication alone was used in 13.8% of patients, whereas surgery alone was performed in one patient (0.6%). No patients were managed with PAIR alone. Symptomatic patients were more frequently treated with surgery plus medication, whereas asymptomatic patients were commonly managed conservatively using a watch-and-wait approach or PAIR with medication. ELISA positivity was more frequently observed among patients undergoing surgery or PAIR procedures. Low hemoglobin levels and leukocytosis were also more common among patients managed with PAIR. Elevated bilirubin levels, observed in 6.3% of patients, occurred predominantly among surgically treated cases (Table 5).

### 3.6. Ultrasound and CT Classification Findings

Ultrasound findings demonstrated that Gharbi Type 3 cysts were most common (28.9%), followed by Type 1 (25.8%) and Type 4 (24.5%). Fewer cases were classified as Type 2 (13.8%) or Type 5 (6.9%). According to the WHO classification, CE1 (25.8%) and CE4 (24.5%) were the most frequent stages, whereas CE2 (17.6%), CE3a (13.8%), CE3b (11.3%), and CE5 (6.9%) were less common.

CT findings showed a similar distribution, with Gharbi Type 3 (29.6%), Type 1 (25.8%), and Type 4 (22.0%) predominating. Slightly higher proportions of CE2 (19.5%) and CE5 (9.4%) cysts were identified by CT compared with ultrasound (Table 6). Overall, ultrasound and CT demonstrated comparable staging distributions, while CT provided additional anatomical detail in selected cases.

### 3.7. Ultrasound Staging and Treatment Selection

Liver hydatid cysts were classified from CE1 to CE5 according to WHO ultrasound criteria, and treatment decisions were guided accordingly. Active CE1 cysts were primarily managed with albendazole therapy, frequently combined with PAIR. CE2 cysts were predominantly treated surgically. Transitional CE3a cysts were mainly treated with albendazole, whereas CE3b cysts were managed using combined surgical and PAIR approaches (Table 7).

Inactive CE4 and CE5 cysts were largely managed using a watch-and-wait strategy. These findings demonstrate the role of ultrasound staging in guiding stage-specific management decisions for liver hydatid cysts.

### 3.8. Association Between WHO Stage and Treatment Modality

Distinct treatment patterns were observed across WHO cyst stages. Active cysts (CE1 and CE2) were mainly treated with PAIR plus medication or surgery plus medication, whereas transitional cysts (CE3a and CE3b) were more frequently managed using surgery combined with medication or medical therapy alone (Table 8).

Inactive cysts (CE4 and CE5) were predominantly managed conservatively using a watch-and-wait approach. Chi-square analysis demonstrated a significant association between WHO cyst stage and treatment modality (χ^2^ = 63.56, df = 15, *p* < 0.001), indicating that management decisions were strongly influenced by ultrasound-based staging.

### 3.9. Agreement Between Ultrasound and CT Classification

Classification of liver hydatid cysts by ultrasound and CT according to both the Gharbi and WHO systems demonstrated highly consistent findings across cyst categories (Table 9). Minor differences were observed in selected categories, particularly Gharbi Type 5 and WHO CE5 cysts. Cohen’s Kappa analysis demonstrated almost perfect agreement between ultrasound and CT classifications (Gharbi κ = 0.959; WHO κ = 0.930), indicating strong concordance between the two imaging modalities for cyst staging.

### 3.10. Treatment Outcomes

Among the 159 patients, the most common treatment modalities were watch-and-wait management (32.0%), surgery combined with medication (29.6%), and PAIR combined with medication (25.2%) (Table 10). Medication alone was mainly used in early or transitional cyst stages, whereas surgery alone was rarely performed. Treatment outcomes varied according to management approach. Surgery combined with medication achieved the highest rate of complete improvement (42 patients, 38.4%), while PAIR combined with medication was associated mainly with partial improvement (34 patients, 30.2%). Patients managed with the watch-and-wait approach largely remained clinically stable without major change during follow-up evaluation (Table 11). A statistically significant association was observed between treatment modality and clinical outcome (*p* < 0.001).

### 3.11. Treatment-Related Complications

Complication rates were low in both treatment groups. The surgical group experienced five complications (3.1%), most commonly wound infection (1.3%), whereas the PAIR group experienced four complications (2.5%), including intraperitoneal leakage (1.3%) and allergic reactions (0.6%). No statistically significant difference in complication rates was observed between surgery and PAIR (χ^2^ = 1.58, *p* = 0.665), indicating comparable safety profiles between the two treatment approaches (Table 12).

## 4. Discussion

Higher prevalence among women and rural residents has also been reported in Khorasan Razavi, Iran (54.9% women and 40.3% rural residents) [13], and was further supported by a systematic review showing increased risk among females (OR 1.38) and rural populations (OR 2.26) [14].

The clinical presentation of liver hydatid cysts in this study was comparable to reports from other endemic settings. Abdominal pain, fever, hepatomegaly, and abdominal tenderness were among the most common findings and were consistent with studies from Nepal [15], South India [16], and a 20-year cohort study [17]. Jaundice was uncommon (3.1%), which is also consistent with previous reports. Only one patient presented with ascites (0.6%), which may have been related to advanced hepatic involvement or another concurrent pathology rather than CE alone. Overall, these findings support the external validity of the observed clinical profile for hepatic CE.

Laboratory findings were also comparable to those reported in other endemic regions. Anemia was present in 28.9% of patients, leukocytosis in 14.5%, and eosinophilia in 22.6%, which is consistent with previous reports describing eosinophilia in approximately 25% of CE cases [18]. Iranian pediatric studies reported higher frequencies of eosinophilia (41%) and leukocytosis (29.2%) [19]. Elevated alkaline phosphatase levels were observed in 22.6% of participants, reflecting hepatic involvement [15]. ELISA serology was positive in 66.0% of cases, supporting its role as a useful adjunct diagnostic tool. However, serological performance may vary depending on cyst stage, cyst burden, antigen release, and cross-reactivity with other parasitic infections, as previously reported [20,21], Therefore, serological findings should be interpreted alongside imaging results. Although ELISA positivity was generally lower in inactive cysts, CE5 cysts showed slightly higher seropositivity than CE4 cysts, possibly due to residual antibody persistence, previous antigen exposure, variation in cyst calcification patterns, or cross-reactivity with other endemic parasitic infections.

This study demonstrated a clear relationship between WHO cyst stage and symptom duration. Early-stage cysts (CE1–CE2) were commonly associated with symptom duration of 8–30 days, whereas transitional and advanced stages (CE3b–CE4) were more frequently associated with prolonged symptoms exceeding 90 days. These findings are consistent with Tamarozzi et al. (2021) and Ley et al. (2025), who reported that chronic or inactive cysts often present with delayed or subtle clinical manifestations [21,22]. Evaluating symptom duration together with cyst stage may therefore improve understanding of disease progression and support timely clinical management.

Management approaches in this study generally followed WHO-IWGE recommendations. The “watch-and-wait” approach (30.8%) was primarily used for inactive cysts (CE4–CE5) and selected uncomplicated cysts, consistent with previous recommendations [23]. PAIR combined with albendazole (25.2%) and surgery plus medication (29.6%) were mainly used for symptomatic, active, or complex cysts, in agreement with current international practice [24,25]. Although surgery is commonly recommended for CE2 cysts, some CE2 patients in our study underwent PAIR combined with medication because of local resource availability, patient preference, cyst accessibility, and individualized clinical decision-making. Patients managed with surgery or PAIR more frequently demonstrated anemia, leukocytosis, and ELISA positivity, suggesting greater cyst activity or disease severity. These findings are similar to reports from Nepal and India [21].

In the present study, ELISA seropositivity was highest in active cyst stages, including CE1 (97.6%) and CE2 (89.3%), while lower positivity was observed in inactive stages, particularly CE4 (17.9%) and CE5 (36.4%). The significant association between cyst stage and ELISA positivity (*p* < 0.001) is consistent with previous reports indicating that active cysts release greater amounts of antigen, thereby stimulating stronger antibody responses [22,26].

The relatively higher seropositivity observed in CE5 compared with CE4 may reflect residual immune responses, previous antigen exposure, variability in calcification patterns, or differences in cyst activity despite imaging evidence of inactivity. These findings further support the combined use of imaging and serology in the diagnosis and management of hepatic CE.

Ultrasound and CT most frequently identified Gharbi Type 3 cysts (28.9% and 29.6%, respectively), while WHO classification demonstrated predominance of CE1 and CE4 cysts. Ultrasound served as the primary imaging modality, whereas CT provided complementary anatomical information in selected complex or calcified cases [27]. Ultrasound-based staging guided treatment selection according to WHO-IWGE and Gharbi classification systems [28], CE1 cysts were mainly managed medically or with PAIR [18,27,28,29], CE2 cysts were commonly treated surgically [18,30], and transitional CE3a–CE3b cysts were managed using combinations of albendazole, PAIR, or surgical intervention depending on cyst characteristics and clinical presentation [29,30].

Inactive CE4 and CE5 cysts were predominantly managed conservatively using a “watch-and-wait” strategy, which is consistent with WHO recommendations for asymptomatic degenerative or calcified cysts [31]. A significant association between WHO cyst stage and treatment selection (χ^2^ = 63.56, *p* < 0.001) further supports the importance of stage-based management approaches and adherence to established WHO-IWGE recommendations [32].

Consistent with findings from Khyber Pakhtunkhwa, where the liver was the most commonly affected organ and substantial gaps in community awareness were identified, our results further support the need for integrated surveillance, early diagnosis, and public health interventions to reduce the burden of cystic echinococcosis in endemic settings [12].

Differential diagnosis of hepatic hydatid cysts includes amoebic liver abscesses, pyogenic (bacterial) liver abscesses, and fungal hepatic lesions. Unlike hydatid cysts, amoebic and pyogenic abscesses commonly present with acute fever, marked inflammatory responses, and imaging findings of irregular thick-walled lesions with surrounding inflammatory changes. Fungal liver lesions are more frequently observed in immunocompromised patients and often appear as multiple small hepatic lesions. In contrast, cystic echinococcosis typically demonstrates characteristic imaging features such as daughter cysts, detached membranes (water-lily sign), hydatid sand, and cyst wall calcification, which facilitate diagnosis and WHO-IWGE staging. Recognition of these distinguishing clinical and radiological characteristics is important for appropriate management and treatment selection [33,34,35].

A recent study by Karatay et al. demonstrated that strain elastography may provide additional diagnostic information when used alongside conventional ultrasonography in patients with hydatid cyst disease. Elastographic assessment may help characterize cyst wall stiffness and internal cyst structure, potentially improving differentiation from other cystic hepatic lesions. Although conventional ultrasonography remains the primary imaging modality recommended for staging according to WHO-IWGE criteria, elastography represents a promising complementary technique that warrants further investigation in endemic settings [36].

The present study demonstrated almost perfect agreement between ultrasound and CT classifications using both Gharbi and WHO systems (Gharbi κ = 0.959; WHO κ = 0.930). [37,38]. These findings indicate that ultrasound provides reliable cyst staging comparable to CT. Previous studies by Pedrosa et al. (2000) [37], El-Tahir et al. (1992) [38], and Beggs (1983) [39] similarly reported high diagnostic utility of ultrasound for detecting daughter cysts, hydatid sand, and inactive lesions. CT remains useful for evaluating anatomical relationships, calcification, and complicated cysts. However, because ultrasound is non-invasive, inexpensive, portable, and widely available, it remains the most practical imaging modality for diagnosis, staging, treatment planning, and follow-up of hepatic hydatid cysts in resource-limited endemic settings, as also supported by Hussain et al. (1985) and Giri et al. (2012) [39,40].

## 5. Study Limitations

This study has several limitations that should be considered when interpreting the findings. First, the study was conducted in a single geographic region of eastern Afghanistan, which may limit the generalizability of the results to other endemic areas with different epidemiological and healthcare characteristics. Second, although the study evaluated treatment outcomes and complications during follow-up, the duration of follow-up was relatively short, limiting assessment of long-term recurrence and treatment durability. Third, inter-observer variability between radiologists was not formally assessed, and no separate reliability analysis for ultrasound readers was performed, although imaging interpretations were independently reviewed. Fourth, detailed analysis of cyst exact anatomical location was not included in the correlation analyses between ultrasound staging, CT findings, and treatment selection, which may have provided additional clinical value for management decisions. Fifth, ELISA serology has inherent limitations related to variable sensitivity and specificity, particularly in inactive cysts and in regions where cross-reactivity with other parasitic infections may occur; therefore, serological findings should be interpreted cautiously alongside imaging results. Sixth, some treatment groups contained small numbers of patients, including surgery alone, which may have reduced the statistical power of subgroup comparisons. Finally, because this was a hospital-based observational study, selection bias may have occurred, as asymptomatic individuals or patients without access to diagnostic facilities may have been underrepresented.

Additionally, the relatively modest sample size may have limited the ability to detect less common imaging patterns and reduced the precision of subgroup analyses. Larger multicenter studies involving broader populations would provide more robust estimates and improve the external validity of the findings.

Furthermore, the cross-sectional design restricted assessment of temporal changes in cyst characteristics and prevented evaluation of disease progression over time. Prospective longitudinal studies would be valuable for examining the evolution of hydatid cyst stages and the long-term impact of imaging-guided management strategies.

The study focused primarily on conventional ultrasonography and computed tomography, which represent the most widely available imaging modalities in routine clinical practice in Afghanistan. Advanced imaging techniques such as magnetic resonance imaging (MRI), strain elastography, and shear-wave elastography were not available within the study setting and therefore could not be evaluated. Future studies incorporating these modalities may provide additional information regarding differential diagnosis and tissue characterization.

Finally, although previous studies have investigated imaging approaches for cystic echinococcosis, the present study was not designed to introduce a novel imaging technique. Rather, its primary contribution lies in providing locally generated evidence from an endemic region where published data remain scarce. Nevertheless, the findings should be interpreted within the context of the existing body of the imaging literature, and further comparative studies involving larger populations and advanced diagnostic technologies are warranted.

Another limitation of this study is that advanced ultrasound techniques such as strain elastography or shear-wave elastography were not available and therefore were not evaluated. These modalities may provide additional information regarding cyst characteristics and differential diagnosis and should be considered in future studies.

## 6. Conclusions

Ultrasound demonstrated high clinical utility for the diagnosis, staging, treatment planning, and follow-up of hepatic hydatid cysts in this endemic setting. The strong agreement observed between ultrasound and CT classifications supports the reliability of ultrasound-based WHO-IWGE staging for guiding stage-specific management decisions. Given its accessibility, affordability, portability, and non-invasive nature, ultrasound should remain the primary imaging modality for hepatic cystic echinococcosis in resource-limited settings such as Afghanistan, while CT should be reserved for complicated, atypical, or anatomically complex cases. The findings of this study further highlight the importance of integrating imaging-based management with improved public health measures, including hygiene promotion, safe food practices, and control of dog-related transmission, to reduce the burden of cystic echinococcosis in endemic communities.

## Figures and Tables

**Table 1:** Socio-demographic, economic, food consumption, water source, and sanitation-related characteristics of patients with hepatic hydatid cysts in Jalalabad, eastern Afghanistan (*n* = 159).

Variable	Categories	*n* (%)	Variable	Categories	*n* (%)
Gender	Male	68 (42.8)	Raw vegetables	Yes	102 (64.2)
	Female	91 (57.2)		Sometimes	42 (26.4)
Age (yrs.)	0–19 years	34 (21.4)		No	15 (9.4)
	20–39 years	80 (50.3)	Water source	Public hand pump	97 (61.0)
	≥40 years	45 (28.3)		Neighbor	42 (26.4)
Marital status	Single	45 (28.3)		Private tube well	18 (11.3)
	Married	98 (61.6)		Piped/private tube well	2 (1.3)
	Widowed	16 (10.1)	Eating style	Hand	114 (71.7)
Residence	Urban	31 (19.5)		Spoon/Both	45 (28.3)
	Rural	128 (80.5)			
Occupation	House worker	64 (40.2)	Hand wash (before meal)	Frequent/Usual	64 (40.2)
	Student	34 (21.4)		Infrequent	95 (59.8)
	Farmer	13 (8.1)	Hand wash (after toilet)	Frequent/Usual	48 (30.2)
	Unemployed	31 (19.5)		Infrequent	111 (69.8)
	Others	17 (10.8)	Toilet type	Flush	9 (5.7)
Education	No education	95 (59.7)		Latrine	48 (30.1)
	Primary/Secondary	45 (28.3)		Open/free area	102 (64.2)
	University	19 (12.0)	Dog ownership	Yes	120 (75.5)
Monthly household income (AFN)	<15,000	126 (79.2)		No	39 (24.5)
	15,000–30,000	30 (18.9)			
	≥30,000	3 (1.9)			

CE: cystic Echinococcosis.

**Table 2:** Clinical and laboratory characteristics of patients with liver hydatid cysts in Jalalabad City, eastern Afghanistan (*n* = 159).

Clinical Characteristics		Lab Investigations	
Variable	*n* (%)	Variable	*n* (%)
**Symptoms**		**Hb level**	
Yes	69 (43.4)	Normal	113 (71.1)
No	90 (56.6)	Low	46 (28.9)
**Abdominal pain**		**WBC**	
Yes	48 (62.8)	Normal	134 (84.3)
No	91 (57.2)	Leukopenia	2 (1.3)
**Abdominal distention**		Leukocytosis	23 (14.5)
Yes	24 (15.1)	**Eosinophilia**	
No	135 (84.9)	Yes	36 (22.6)
**Nausea/Vomiting**		No	123 (77.4)
Yes	37 (23.3)	**Serum bilirubin**	
No	122 (76.7)	Normal	149 (93.7)
**Jaundice**		High	10 (6.3)
Yes	5 (3.1)	**Alkaline phosphatase**	
No	154 (96.9)	Normal	123 (77.4)
**Weight loss**		High	36 (22.6)
Yes	15 (9.4)	**Stool exam intestinal parasites**	
No	144 (90.6)	Positive	49 (30.8)
**Fever**		Negative	104 (65.4)
Yes	46 (29)	Not done	6 (3.8)
No	113 (71)	**ELISA**	
**Abdominal tenderness**		Positive	105 (66.0)
Yes	67 (42.1)	Negative	54 (34.0)
No	92 (57.9)		
**Hepatomegaly**			
Yes	20 (12.6)		
No	139 (87.4)		
**Ascites**			
Yes	1 (0.6)		
No	158 (99.4)		

**Table 3:** Symptom duration according to WHO stage classification among symptomatic patients with liver hydatid cysts in Jalalabad City, eastern Afghanistan (*n* = 69).

Stage	0–7 Days Very Acute	8–30 Days (Acute to Sub-Acute)	31–90 Days (Sub-Acute to Chronic)	>90 Days (Chronic)
CE1	4 (17.4%)	16 (69.6%)	3 (13.0%)	0 (0.0%)
CE2	1 (5.9%)	12 (70.6%)	4 (23.5%)	0 (0.0%)
CE3a	0 (0.0%)	3 (23.1%)	8 (61.5%)	2 (15.4%)
CE3b	0 (0.0%)	0 (0.0%)	2 (20.0%)	8 (80.0%)
CE4	0 (0.0%)	0 (0.0%)	0 (0.0%)	6 (100.0%)
CE5	0 (0.0%)	0 (0.0%)	0 (0.0%)	0 (0.0%)
Total	5 (7.2%)	31 (44.9%)	17 (24.6%)	16 (23.2%)

**Table 4:** ELISA serology results according to ultrasound-based WHO-IWGE cyst classification.

Stage	Positive (*n*)	Positive (%)	Negative (*n*)	Negative (%)	Total (*n*)
CE1	40	97.6%	1	2.4%	41
CE2	25	89.3%	3	10.7%	28
CE3a	16	72.7%	6	27.3%	22
CE3b	13	72. 2%	5	27.8%	18
CE4	7	18.0%	32	82.0%	39
CE5	4	36.4%	7	63.6%	11
Total	105	66.04%	54	33.9%	159

Chi-square test showed a significant association between WHO cyst stage and ELISA seropositivity (*p* < 0.001).

**Table 5:** Clinical and Laboratory characteristics across the treatment methods of the liver hydatid cyst disease (*n* = 159).

Categories	Clinical Characteristics		Laboratory									Total (%)
			ELISA Serology		Hemoglobin (g/dL)		WBC Count ×10^3^/µL			Serum Bilirubin mg/dL		
	Symptomatic	Asymptomatic	Negative	Positive	Normal	Low	Normal	Leukopenia	Leukocytosis	Normal	High	
Wait and Watch	6	43	38	11	37	12	44	1	4	47	2	51 (32.0%)
Medication	9	13	8	14	15	7	16	1	5	21	1	20 (12.6%)
Surgery alone	0	1	1	0	1	0	0	0	1	1	0	1 (0.6%)
Surgery with medication	35	12	3	44	36	11	35	0	12	40	7	47 (29.6%)
PAIR with medication	19	21	4	36	24	16	39	0	1	40	0	40 (25.2%)

ELISA: enzyme-linked immunosorbent assay.

**Table 6:** Classification of liver hydatid cysts according to ultrasound and CT findings using the Gharbi and WHO-IWGE systems (*n* = 159).

Ultrasound Findings					
Gharbi Classification	Frequency	Percentage	WHO Classification	Frequency	Percentage
Type 1	41	25.8%	CE1	41	25.8%
Type 2	22	13.8%	CE2	28	17.6%
Type 3	46	28.9%	CE3a	22	13.8%
Type 4	39	24.5%	CE3b	18	11.3%
Type 5	11	7.0%	CE4	39	24.5%
			CE5	11	7.0%
**CT Findings**					
**Gharbi Classification**	**Frequency**	**Percentage**	**WHO Classification**	**Frequency**	**Percentage**
Type 1	41	25.8%	CE1	41	25.8%
Type 2	21	13.2%	CE2	31	19.5%
Type 3	47	29.6%	CE3a	21	13.2%
Type 4	35	22.0%	CE3b	16	10.1%
Type 5	15	9.4%	CE4	35	22.0%
			CE5	15	9.4%

**Table 7:** WHO Ultrasound Staging and Treatment Selection.

WHO Cyst Stage (US)	Typical Characteristics	Expected Treatment Approach (Based on WHO Guidelines)	Actual Treatment Choices
CE1 (Unilocular, active cyst)	Fluid-filled, early stage	Albendazole ± PAIR or Surgery	Mostly Albendazole and PAIR are used
CE2 (Multivesicular, active)	Multiple daughter cysts, high pressure	Surgery is preferred due to the complexity	Many underwent Surgery
CE3a (Transitional—detached membrane)	“Water lily sign”	PAIR or Albendazole	Mainly Albendazole, some PAIR
CE3b (Transitional—daughter cysts)	Solid matrix + daughter cysts	Often Surgery or PAIR	Some Surgery and PAIR used
CE4 (Inactive)	Heterogeneous, degenerating cyst	Observation (“watch and wait”)	Largely managed by “Watch and Wait”
CE5 (Calcified, inactive)	Calcified wall, dead cyst	No treatment (“watch and wait”)	Mostly “Watch and Wait”

**Table 8:** Association between ultrasound-based WHO-IWGE cyst stage and treatment modality.

WHO Cyst Stage	Medication	PAIR with Medication	Surgery Alone	Surgery with Medication	Watch-and-Wait	Total
CE1—Unilocular, active cyst	8	18	0	14	1	41
CE2—Multivesicular, active	3	14	0	11	0	28
CE3a—Transitional, detached membrane (“Water lily sign”)	2	8	0	12	0	22
CE3b—Transitional, daughter cysts	7	0	0	10	1	18
CE4—Inactive, degenerating cyst	0	0	0	0	39	39
CE5—Calcified, inactive cyst	0	0	1	0	10	11
Total	20	40	1	47	51	159

Chi-square test demonstrated a significant association between WHO cyst stage and treatment modality (χ^2^ = 63.56, df = 15, *p* < 0.001). PAIR: puncture, aspiration, injection, and re-aspiration.

**Table 9:** Comparison of ultrasound and CT findings by Gharbi and WHO classifications of liver hydatid cyst patients (*n* = 159).

	Ultrasound		CT Scan		Cohen’s Kappa
Classification	Frequency (*n*)	Percentage (%)	Frequency (*n*)	Percentage (%)	
Gharbi Type 1	41	25.8%	41	25.8%	Kappa = 0.930
Gharbi Type 2	22	13.8%	21	13.2%	
Gharbi Type 3	46	28.9%	47	29.6%	
Gharbi Type 4	39	24.5%	35	22.0%	
Gharbi Type 5	11	7%	15	9.4%	
WHO CE 1	41	25.8%	41	25.8%	Kappa = 0.930
WHO CE 2	28	17.6%	31	19.5%	
WHO CE 3a	22	13.8%	21	13.2%	
WHO CE 3b	18	11.3%	16	10.1%	
WHO CE 4	39	24.5%	35	22.0%	
WHO CE 5	11	7%	15	9.4%	

**Table 10:** Treatment distribution according to ultrasound-based WHO classification among patients with hepatic hydatid cysts (*n* = 159).

Categories	WHO Classification						
	CE1	CE2	CE3a	CE3b	CE4	CE5	Total (%)
Wait and Watch	1	0	0	1	39	10	51 (32.0)
Medication	8	3	2	7	0	0	20 (12.6)
Surgery alone	0	0	0	0	0	1	1 (0.6)
Surgery with medication	14	11	12	10	0	0	47 (29.6)
PAIR with medication	18	14	8	0	0	0	40 (25.2)
Total (%)	41 (25.8%)	28 (17.6%)	22 (13.8%)	18 (11.3%)	39 (24.5%)	11 (7%)	159 (100%)

**Table 11:** Treatment outcomes according to management modality among patients with hepatic hydatid cysts in Jalalabad, eastern Afghanistan (*n* = 159).

Treatment Outcome	Treatment Methods					
	Wait and Follow up Monthly	Medication	Surgery Alone	Surgery + Medication	PAIR + Medication	Total
Complete cure	0	12	1	42	5	61(38.4%)
Partial cure	0	8	0	5	34	48 (30.2%)
Lost to follow-up	1	0	0	0	1	2 (1.2%)
Unchanged	50	0	0	0	0	48 (30.2%)
Total	51 (32.0%)	20 (12.6%)	1 (0.6%)	47 (29.6%)	40 (25.2%)	159 (100%)
*p* value (x^2^)						<0.001

**Table 12:** Post-treatment complications among patients undergoing surgery or PAIR for hepatic hydatid cysts in Jalalabad, eastern Afghanistan (*n* = 159).

Surgery Procedure		PAIR	
Type of Complications	Frequency (%)	Type of Complications	Frequency (%)
Wound Infection	2 (1.3%)	Allergic reaction	1 (0.6%)
Biliary Fistula	1 (0.6%)	Peri hepatic hemorrhage	1 (0.6%)
Hepatic Abscess	1 (0.6%)	Intra peritoneal leakage	2 (1.3%
Incisional Hernia	1 (0.6%)		
Total number (%)	5 (3.1%)	Total number (%)	4 (2.5%)

Chi-square statistic: 1.58, *p*-value: 0.665, Degrees of freedom: 3.

## Data Availability

Data are available from the corresponding author on reasonable request.

## Abbreviations

AE	Alveolar echinococcosis
CBC	Complete blood count
CE	Cystic echinococcosis
CE1–CE5	World Health Organization cyst stages 1 to 5
CEUS	Contrast-enhanced ultrasound
CT	Computed tomography
df	Degrees of freedom
*E. granulosus*	*Echinococcus granulosus*
*E. multilocularis*	*Echinococcus multilocularis*
ELISA	Enzyme-linked immunosorbent assay
IgG	Immunoglobulin G
IBM	International Business Machines (IBM SPSS software)
IRB	Institutional Review Board
IWGE	Informal Working Group on Echinococcosis
MRI	Magnetic resonance imaging
PAIR	Puncture, aspiration, injection, re-aspiration
PET	Positron emission tomography
SPSS	Statistical Package for the Social Sciences
SWE	Shear wave elastography
US	Ultrasound
USG	Ultrasonography
WHO	World Health Organization
WHO CE	World Health Organization cystic echinococcosis classification
WHO-IWGE	World Health Organization Informal Working Group on Echinococcosis
Z	Z-score
